# Supplementary material for: Shaofu Zhuyu Decoction for Treating Endometriosis: A Systematic Review and Meta-Analysis
Source: Pharmaceuticals (Basel). 2025 Aug 29;18(9):1296. doi: 10.3390/ph18091296 (PMC12472636; doi:10.3390/ph18091296)
Supplement: Supplementary file 1 [file pharmaceuticals-18-01296-s001.zip › pharmaceuticals-3824353-supplementary/Additional File S2. Search terms used in each database.pdf]

**Supplement 2. Search terms used in each database and results**

**Medline via PubMed (15.03.2025.)**

| | Searches |
|---|---|
| #1 | "Shaofu Zhuyu decoction"[TIAB] OR "Shaofu Zhuyu Tang"[TIAB] OR "Shaofu Zhuyu formula"[TIAB] |
| #2 | Endometriosis[MH] |
| #3 | "randomized controlled trial"[PT] OR "controlled clinical trial"[PT] OR randomized[TIAB] OR placebo[TIAB] OR "drug therapy"[SH] OR randomly[TIAB] OR trial[TIAB] OR groups[TIAB] |
| **#4** | **#1 AND #2 AND #3** |

**Embase via Elsevier (15. 03.2025.)**

| | Searches |
|---|---|
| #1 | "Shaofu Zhuyu decoction"/exp OR "Shaofu Zhuyu decoction":ab,ti OR "Shaofu Zhuyu Tang"/exp OR "Shaofu Zhuyu Tang":ab,ti OR "Shaofu Zhuyu formula"/exp OR "Shaofu Zhuyu formula":ab,ti |
| #2 | "endometriosis"/exp OR "endometriosis":ab,ti |
| #3 | "crossover procedure":de OR "double-blind procedure":de OR "randomized controlled trial":de OR "single-blind procedure":de OR (random* OR factorial* OR crossover* OR cross NEXT/1 over* OR placebo* OR doubl* NEAR/1 blind* OR singl* NEAR/1 blind* OR assign* OR allocat* OR volunteer*):de,ab,ti |
| **#4** | **#1 AND #2 AND #3** |

**CENTRAL (15. 03.2025.)**

| | Searches |
|---|---|
| #1 | ("Shaofu Zhuyu decoction" OR "Shaofu Zhuyu Tang" OR "Shaofu Zhuyu formula"):ti,ab,kw |
| #2 | MeSH descriptor: [Endometriosis] explode all trees |
| #3 | "Endometriosis":ti,ab,kw |
| **#4** | **(#1 AND (#2 OR #3)) in Trials** |

**Chinese medical databases – CNKI , Wanfang Data (15.03.2025.)**

| | Searches |
|---|---|
| #1 | 少腹逐瘀汤 OR "Shaofu Zhuyu decoction" OR "Shaofu Zhuyu Tang" OR "Shaofu Zhuyu formula" |
| #2 | 子宫内膜异位症 OR endometriosis |
| #3 | 随机 OR 临床试验 OR randomization OR random |
| **#4** | **#1 AND #2 AND #3** |

**Korean medical databases – ScienceON, the Korean Traditional Knowledge Portal, KoreaMed, the Oriental Medicine Advanced Searching Integrated System (OASIS), the Research Information Sharing Service, and the National Library of Korea (15.03.2025.)**

| | Searches |
|---|---|
| #1 | "Shaofu Zhuyu decoction" OR "Shaofu Zhuyu Tang" OR "Shaofu Zhuyu formula" OR 소복축어탕 |
| #2 | 자궁내막증 OR Endometriosis |
| #3 | 무작위 OR 임상연구 OR randomization OR random |
| **#4** | **#1 AND #2 AND #3** |
